# Co-infection dynamics of COVID-19 and HIV/AIDS

**DOI:** 10.1038/s41598-023-45520-6

**Published:** 2023-10-27

**Authors:** Tesfaneh Debele Batu, Legesse Lemecha Obsu, Chernet Tuge Deressa

**Affiliations:** 1 Department of Applied Mathematics, Adama Science and Technology University, Adama, Ethiopia; 2 Department of Mathematics, Jimma University, Jimma, Ethiopia

**Keywords:** Computational biology and bioinformatics, Diseases, Mathematics and computing

## Abstract

Although there are many results that can be used to treat and prevent Coronavirus Disease 2019 (COVID-19) and Human Immunodeficiency Virus (HIV), these diseases continue to be public health concerns and cause socioeconomic consequences. Following compromised immunity, COVID-19 is considered to be a challenge for people with HIV. People with advanced HIV are considered a vulnerable population at high risk in several case studies that discuss COVID-19 and HIV co-infection. As there is no cure for HIV and there is a chance of contracting COVID-19 again, co-infection continues to pose a problem. The purpose of this study is to investigate the impact of intervention strategies and identify the role of different parameters in risking people living with HIV to death when they get infected with COVID-19. This is achieved through the development and rigorous analysis of a mathematical model that considers a population at risk of death due to COVID-19 and HIV. The model formulation provides a detailed explanation of the transmission dynamics of COVID-19 and HIV co-infection. The solution’s invariant region, positivity, and boundedness were established. The reproduction numbers of the sub-models and the co-infection model were determined. The existence and stability of equilibria, including backward bifurcation for the COVID-19 sub-model, were examined. The epidemiological significance of backward bifurcation is that the condition ${\mathscr {R}}_0$ less than 1 for eliminating COVID-19, though necessary, is no longer sufficient. Parametric estimation and curve fitting were performed based on data from Ethiopia. Numerical simulations were employed to support and clarify the analytical findings and to show some parameter effects on COVID-19 and HIV co-infection. Accordingly, the simulations indicated that parameters $\gamma _c$, $\gamma _h$, $\epsilon$, and $\kappa$, related to HIV patients’ exposure to other diseases and the increase in infectiousness, have a positive role in increasing the number of co-infections. On the other hand, an increase in COVID-19 vaccination ($\xi$) shows the suppression of co-infection cases. In addition, treating co-infected individuals for COVID-19, increasing treatment rates $\alpha$ and $\varphi$, reduces the death risk of HIV-infected individuals due to the co-infection burden. It was implied that improving vaccine delivery programs and other medical interventions have important contributions to lowering the risk of COVID-19 infection-related fatalities in HIV patients.

## Introduction

HIV has emerged as one of the most significant single causes of death and suffering on the globe. A systematic analysis of the global disease burden conducted in^1^ showed that HIV was the eleventh leading factor in the burden of diseases in 2019. In particular, HIV was the ninth and second leading cause of the disease worldwide in the age groups 10–24 and 25–49, respectively. This result shows that HIV remains a challenge to be confronted by people around the world. With nearly 36.3 million (27.2–47.8 million) lives claimed so far, HIV is recognized as a significant issue in global public health by the WHO global HIV program^2^. According to this program’s estimates, in 2020, 37.7 million (30.2–45.1 million) people were living with HIV, 1.5 million (1–2 million) had become newly infected over the previous year, and 800000 (480000-1 million) people worldwide died from HIV-related causes.

HIV compromises immunological function by attacking cells (CD4^+^ T-cells) that play an essential role in protecting the host from pathogens^3^. Once HIV destroys these cells, it becomes harder for the body to fight off other infections. Unprotected sexual contact, hypodermic needles, contaminated blood transfusions, and breastfeeding are the most common ways in which HIV is transmitted^4^. The most common method of HIV transmission is still heterosexual contact^5^. The infectiousness of the HIV-infected partner determines whether HIV may be transmitted; larger viral loads in the disease’s later stages are linked to a higher risk of transmission^6^. As HIV advances, the infection is usually called AIDS, Acquired Immune Deficiency Syndrome. Although it cannot be permanently prevented, prevention mechanisms such as faithfulness, protection, and abstinence are the main methods used to combat the disease. These methods depend primarily on the level of behavioral change in the population and the antiretroviral therapy (ART) offered to those infected^3^. ART therapy enhances health, lengthens life, and significantly lowers the risk of HIV transmission^6^.

Recently, COVID-19 has been considered a primary worldwide public health concern. It was first detected in Wuhan, China, in December 2019. Since the onset of the epidemic, 764,154,749 people have been infected, and 6, 914, 938 people have lost their lives up to April 20, 2023^7^. Preexisting comorbidities, such as HIV, cardiovascular diseases, hypertension, chronic obstructive pulmonary disease, diabetes, and malignancies, are considered primary factors in worsening the impact of the pandemic^8–15^. In particular, since COVID-19’s emergence, several reports of COVID-19 and HIV co-infection have been obtained. As of April 29, 2021, 24 countries had contributed clinical data on people living with HIV (PLHIV) to the WHO Global COVID-19 Clinical Data Platform^16^. This global sample showed that 9.2% (15522/168649) of cases hospitalized with expected or proven COVID-19 were HIV positive, and 96.1% (14914/15522) of the PLHIV included in the analysis were from the WHO African region, with 94.6% (14682/15522) of cases reported from South Africa alone. From the global sample, the prevalence of HIV was found to be a significant independent risk factor for severe or critical COVID-19 disease at hospitalization and in-hospital death.

After the development of coronavirus vaccines, governments have vaccinated their citizens as an effective strategy to halt the COVID-19 pandemic. The WHO has validated several COVID-19 vaccines for use. As of April 8, 2022, the WHO has a list of ten COVID-19 vaccines that have met the necessary criteria for safety and efficacy^17^. Despite some single-dose vaccines, the majority of these vaccines require at least 2 doses, and taking COVID-19 full-dose provides strong protection against serious illness, hospitalization, and death due to the COVID-19 disease. As of April 29, 2023, a total of 5,105,854,153 individuals were fully vaccinated with the last dose of the primary series globally^7^. Of these, 37,465,340 people live in Ethiopia, i.e., 32.59% of the total population, were fully vaccinated. Because they seem to be at a greater risk of severe consequences and mortality from COVID-19 than other people, the World Health Organization advises that PLHIV should be a designated priority group in the national COVID-19 vaccination program, regardless of their CD4 level^18^. In addition to vaccination, other methods, such as hand hygiene compliance, early diagnosis, prevention of public contact, contact tracing, voluntary home quarantine, and travel restrictions were considered to decrease human-to-human transmission of COVID-19 disease^19^.

Modeling a disease’s transmission dynamics can assist researchers in making future predictions and taking precautions to prevent its transmission to the maximum number of individuals. In addition, the specified tools help to find out strategies for controlling or eliminating the disease from the community. Papers^20–22^ and^23^, among many others, describe the co-dynamics of diseases and suggest various techniques to mitigate the impact of co-infection on the host’s health and the spread of diseases in society. In relation to COVID-19, several studies have been conducted on the co-dynamics of the virus with other diseases. The authors in^24–30^ proposed and analyzed a deterministic model for COVID-19 co-infection. In^24^, a compartmental model of cholera-COVID-19 co-infection was investigated by considering the transmission dynamics of both diseases. Using an optimal control strategy,^25^ studied malaria and COVID-19 co-dynamics. In^26^, a mathematical model was developed to explain the impact of diabetes on COVID-19 complications and vice versa. It was found that decreasing infection peaks are associated with declining re-infection rates of those who have recovered from COVID-19 and that preventing infection in comorbid susceptibles is the most cost-effective strategy for controlling the disease. An in-depth analysis of COVID-19 and TB co-infection dynamics has been provided in^27^. Results from the study show that a significant reduction in contact rates combined with an increase in treatment leads to a reduction in the spread of TB and COVID-19 co-infection. In contrast to many other co-infection models that overlook the impact of incident co-infection, Omame et al.^28,29^ suggested a novel mathematical model for the co-dynamics of SARS-CoV-2 with Hepatitis B and Zika. In their work, backward bifurcation analysis show that the dynamics of the sub-model do not necessarily drive or impact the dynamics of the whole co-infection model when a model incorporates incident co-infection with both diseases. Through numerical simulations, the studies show the substantial impact of SARS-CoV-2 prevention in decreasing the burden of co-infections with Hepatitis B and Zika and vice versa. To assess the impacts of COVID-19 on the dynamics of Zika, Dengue, and Chikungunya as well as the reverse, a mathematical model for COVID-19, Zika, Chikungunya, and dengue co-dynamics is developed and explored in^30^. In this paper, for the purpose of preventing the co-circulation of the diseases, time-dependent controls are taken into account and assessed utilizing Pontryagin’s principle. The simulations of the optimized system show that implementing a combined prevention strategy leads to a significantly high impact compared to solely focusing on preventing one particular disease. These highlights the importance of a holistic approach when implementing control measures to combat COVID-19, Zika, Dengue, and Chikungunya.

Although several studies are reported on co-infection of various disease, literature reveals that the study on HIV and COVID-19 co-infection models remain few^31–34^. The authors in^31^ proposed a within-host SARS-CoV-2/HIV co-infection model and and show that when SARS-CoV-2/HIV co-infected patients have weak CD4+ T-cell immunity, more productively infected epithelial cells and SARS-CoV-2 particles are produced. Moreover, their findings indicate that lowering the severity of SARS-CoV-2 infection in HIV patients can be achieved by raising the death rate of infected epithelial cells during the latency period. A fractional order model for Dual Variants of COVID-19 and HIV co-infection via Atangana-Baleanu derivative is given in^32^. Based on the analytical and numerical results, the authors of^32^ argue that the COVID-19 vaccination has a significant impact on the co-infection dynamics of HIV and COVID-19 variants, leading to a decrease in prevalence as vaccination rates increase. Particularly, the study reported in^33^ and^34^ employ systems of ordinary differential equations to describe the transmission dynamics of COVID-19 and HIV. In both^33^ and^34^, eight compartments were taken into consideration that characterize the co-dynamics of COVID-19 and HIV. Various parametric values were used in^34^ to analyze their effects on the co-infection. On the other hand, to enable a holistic understanding of the impact of implementing one strategy over the other, time-dependent control strategies were introduced in^33^. The result of these studies show that prevention and treatment strategies play a significant role in reducing the burden of the disease. In spite of all of their contributions, COVID-19 re-infection^33^ and stages associated with HIV and COVID-19 co-infection are still not taken into account^33,34^.

As we continue to navigate the pandemic, reinfection with COVID-19 has become increasingly relevant. There have been documented cases of individuals becoming infected with COVID-19 more than once, with some experiencing more severe symptoms. As we work to mitigate the spread of COVID-19 and HIV co-infection, it’s important to keep in mind the potential for reinfection. Moreover, it’s important to recognize the significance of including advanced HIV patients in co-dynamics studies of HIV and COVID-19. This is because, HIV response continues to face a persistent problem with advanced HIV disease^35^ and co-infection with COVID-19 raises the complexity of this problem. People at risk of death due to COVID-19 and HIV/AIDS co-infection are also needed to be considered, aiming at determining intervention strategies to reduce the loss of lives among people living with HIV. Motivated by the aforementioned cases and the scarcity research works in this topic, we intended to propose and analyze mathematical model to see the effect of different parameters in HIV and COVID-19 co-infection. For this purpose, we introduce a new compartmental model wherein the entire population is subdivided into nine distinct compartments: susceptible, vaccinated, COVID-19 infected, recovered, HIV infected, HIV-positive population with advanced stage of the disease, HIV and COVID-19 infected, people with advanced HIV and infected with COVID-19, and at risk to death population due to the co-infection. We consider reinfection with COVID-19 and the proportion of the vaccinated population protected by vaccine. Analytical as well as numerical results are used to realize the role of COVID-19 and HIV transmission, HIV transmissibility, immunosuppression due to HIV, vaccination and treatments on the co-infected population.

The remaining part of the paper is organized as follows: Establishing the co-infection model is the primary concern of Section “Model formulation”. The positivity and boundedness of the solution are discussed in Section “Results”. The detailed analyses for sub and co-infection models are given in Sections “Analysis of the sub models” and “Analysis of the co-infection model”, respectively. Based on data from Ethiopia, parameter estimation is presented in Section “Parametric estimation”. The analytical findings are supported by numerical simulations in Section “Numerical simulation and results”. Determining the effects of some parameters on COVID-19 and HIV co-infection is also the concern of this section. Sections “Discussion” and “Conclusion” are devoted to the discussion and conclusions, respectively.

## Methods

The method to carry out the research relies on the objectives of the paper and the availability of biological knowledge on the pathogenesis of the infection and the epidemiology of the diseases. To come up with these bases, we formulate compartmental models that take several assumptions into account. The mode is formulated based on nonlinear systems of differential equations to describe the dynamics of COVID-19 and HIV/AIDS as time progresses. The existing mathematical theories are used to analyze the model’s basic properties: positivity, boundedness, existence, and uniqueness. Local and global stability analyses are performed using linearization and global stability results. The model parameters are estimated so as to make reliable quantitative predictions. Numerical simulations are performed with the help of Python 3.9 and are used as a tool for validating mathematical descriptions of the situation and to obtain predictions that could be compared with observations recorded.

## Model formulation

In formulating co-infection model, the total population at time *t*, denoted by *N*(*t*), is categorized into different compartments based on their epidemiological state: susceptible(*S*), COVID-19 fully vaccinated (*V*), COVID-19 infected individuals ($I_C$), individuals recovered from COVID-19 (*R*), HIV infectious but asymptomatic ($I_H$), individuals with advanced HIV disease ($I_A$), HIV and COVID-19 infected ($I_{HC}$), HIV and COVID-19 co-infected with clinical signs of AIDS ($I_{AC}$) and people at high risk of death due to co-infection ($D_{HC}$).

**Figure 1:**
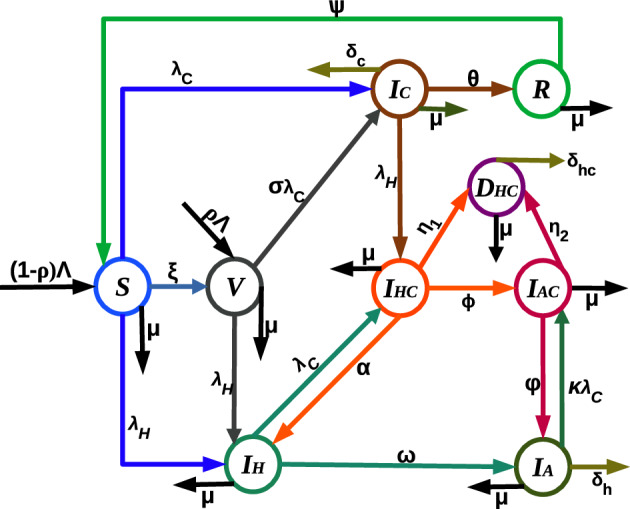
Model flow diagram for co-infection model.

The flow through the compartmental subpopulations is described as follows. A fraction of $(1-\rho ) \Lambda$ human population is recruited in the susceptible class, where $\rho$ is the proportion of vaccinated population and $\Lambda$ is a recruitment rate of susceptible population. The remaining human population, $\rho \Lambda$, joins the vaccinated class. Loss of immunity after $\frac{1}{\psi }$ period of time in recovered class raises susceptible class. Following effective contact with COVID-19 and HIV infected individual, a susceptible may acquire COVID-19 and HIV diseases at rate of $\lambda _C$ and $\lambda _H$, respectively. The rate at which susceptible become COVID-19 infected from individuals with active COVID-19( $\lambda _C$), is $\lambda _C = \gamma _c \frac{I_C+ I_{HC} + I_{AC}}{N-D_{HC}},$ where $\gamma _c$ stands for the rate of transmission of the COVID-19 disease. Due to severe health conditions, it was assumed that individuals at high risk of death ($D_{HC}$) are under follow-up and do not involve in the transmission of both diseases. The rate at which susceptible individuals acquire HIV infection from individuals with active HIV($\lambda _H$), is $\lambda _H = \gamma _h \frac{I_H + I_{HC}+ \varepsilon (I_A + I_{AC})}{N-D_{HC}} ,$ where $\gamma _h$ stands for the rate of transmission of the HIV disease. Higher viral loads in the later stages of the disease increase the HIV transmission probability. So, it was assumed that individuals in ($I_{AC}$) class has transmission rate $\beta \varepsilon$, where $\varepsilon \ge 1$ is a modification parameter for the assumption that a individuals with advanced HIV are more likely to transmit the disease. Natural death decreases susceptible and other subpopulations at a rate of $\mu$. It is assumed that vaccination is applied only to healthy individuals, so only susceptible individuals get vaccinated at a rate of $\xi$. There are findings which argue that COVID-19 vaccinated individuals can become infected even though they have been vaccinated. For this purpose the infection of vaccinated individuals happens at a reduced transmission rate $\sigma \gamma _c$, where $0 \le \sigma \le 1$ is the reduction coefficient. Here, if $\sigma = 0$, vaccinated individuals cannot get infected, and the vaccine is perfect. If $\sigma = 1$, vaccinated individuals get infected like susceptible individuals, and immunization plays no protective role. The rate of change of the COVID-19 infected population is increased due to transfer from the susceptible and vaccinated classes at the rate of $\lambda _C$ and $\sigma \lambda _C$ respectively. This population decreases as infectious individuals are recovered at the rate $\theta$ or die due to the disease at the rate of $\delta _c$. The rate of change of the HIV infected population is generated at a rate of $\lambda _H$ from the susceptible and vaccinated population. Interacting with a COVID-19 infected person at rate of $\lambda _C$ reduces the number of population in $I_H$ class. Members of $I_{A}$ transferred from $I_H$ class at a rate of $\omega$ become infected with COVID-19 at a rate $\kappa \lambda _C$. Modification parameter $\kappa \ge 1$ accounts for the fact that there is an increased risk of getting COVID-19 for someone already infected with HIV, due to the vulnerability of the immune system. Moreover, they may lost their life due to HIV at the rate of $\delta _h$. The number of populations in $I_{HC}$ and $I_{AC}$ classes, following the burden of the co-infection, transferred to $D_{HC}$ family at a rate $\eta _1$ and $\eta _2$, and recovered from COVID-19 at a rate $\alpha$ and $\varphi$, respectively. Co-infected individuals risked to death may lost their life at a rate $\delta _{hc}$. The description of the remaining parameters can be seen from Table 1. Bringing the aforementioned assumptions together leads to the set of nonlinear ordinary differential equations given in (1). The schematic representation of model (1) is illustrated in Fig. 1. It schematically represents the epidemiology of COVID-19 and HIV/AIDS co-infection. Circles are used to depict different diseases stages, and arrows show how people advance from one stage to the next.

The following system of differential equations describe the co-infection dynamics of COVID-19 and HIV/AIDS.

1 $$\begin{aligned} {\left\{ \begin{array}{ll} &{}S^\prime = (1-\rho ) \Lambda + \psi R - \left( D_1 + \lambda _C +\lambda _H \right) S,\\ &{}V^\prime = \rho \Lambda + \xi S - \left( \mu + \sigma \lambda _C + \lambda _H\right) V, \\ &{}I_C^\prime = \lambda _C S + \sigma \lambda _C V - \left( D_2 + \lambda _H \right) I_C, \\ &{}R^\prime = \theta I_C - D_3 R, \\ &{}I_H^\prime = (S + V) \lambda _H + \alpha I_{HC} - \left( D_4 + \lambda _C\right) I_H,\\ &{}I_A^\prime = \omega I_H + \varphi I_{AC} - \left( D_5 + \kappa \lambda _C\right) I_A,\\ &{}I_{HC}^\prime = \lambda _C I_H + \lambda _H I_C - D_6 I_{HC},\\ &{}I_{AC}^\prime = \phi I_{HC} + \kappa \lambda _C I_A - D_7 I_{AC},\\ &{}D_{HC}^\prime = \eta _1 I_{HC} + \eta _2 I_{AC} - D_8 D_{HC}, \end{array}\right. } \end{aligned}$$

where $$D_1= \mu +\xi$$, $$D_2 = \theta + \mu + \delta _c$$, $$D_3=\mu +\psi$$, $$D_4=\omega + \mu$$, $$D_5 = \mu + \delta _h$$, $$D_6 = \alpha + \phi + \mu + \eta _1$$, $$D_7 = \varphi + \mu + \eta _2$$ and $$D_8 = \delta _{hc} + \mu$$.

**Table 1:** Description of parameters of the model system (1).

Parameters	Description
$\Lambda$	Recruitment rate for susceptible
$\rho$	Fraction of recruitment to the vaccinated class
$1-\sigma$	COVID-19 vaccine efficacy
$\xi$	Vaccination rate for susceptible individuals
$\epsilon , \kappa$	Modification parameters
$\gamma _c$	Effective transmission rate of COVID-19
$\gamma _h$	Effective transmission rate of HIV
$\theta$	The recovery rate of the COVID-19-infected class
$\mu$	Natural death rate
$\delta _c$	Death due to COVID-19 disease
$\psi$	Rate of loosing immunity after COVID-19 recovery
$\omega$	HIV progression rate for individuals in $I_{H}$
$\delta _h$	HIV induced death rate
$\eta _1, \ \eta _2$	Rate at which co-infected individuals risked to death
$\alpha$	Recovery rate from COVID-19 for $I_{HC}$
$\varphi$	Recovery rate from COVID-19 for $I_{AC}$
$\delta _{hc}$	Co-infection induced death rate
$\phi$	HIV progression rate for individuals in $I_{HC}$

## Results

Since model system (2) involves a human population, all solutions must be positive and bounded and these solutions are realized in a positively invariant region. The positivity of the solutions and the positively invariant region will be investigated through Theorem 4.1 under the presumption that all parameters are positive.

### Theorem 4.1

The solutions of system (1) with positive initial data remain positive for all $t > 0$. Furthermore, the biologically feasible region

$$\begin{aligned} \Theta =\{(S, V, I_C, R, I_H, I_A, I_{HC}, I_{AC}, D_{HC}) \in {\mathbb {R}}^9 :0 \le \ N \le \Lambda /\mu \} \end{aligned}$$

is positively invariant and attracting for system (1).

### Proof

Equations in (1) points inward on the boundary ${\mathbb {R}}_+^9\setminus \{{\textbf{0}}\}$. For instance if $S = 0$, then,

$$\begin{aligned} \frac{dS}{dt} = (1-\rho ) \Lambda + \psi R > 0. \end{aligned}$$

Applying the same procedure, we can show that the remaining state variables are also positive $\forall t > 0$.

Furthermore, adding all the equations of the model system (1), total human populations satisfy the following equation,

$$\begin{aligned} \frac{dN}{dt} \le \Lambda - \mu N. \end{aligned}$$

Integrating both sides from 0 to *t*, we have

$$\begin{aligned} N(t)-N(0) \le -\mu \int _{0}^{t}\left( N(s) - \frac{\Lambda }{\mu }\right) ds. \end{aligned}$$

If $0 \le N(0) \le \frac{\Lambda }{\mu }$, from Gronwall inequality, we can deduce that

$$\begin{aligned} 0 \le N \le \Lambda /\mu . \end{aligned}$$

Therefore, all solutions in ${\mathbb {R}}_+^9$ are drawn to the region $\Theta$. $\square$

The implication of *Theorem *4.1 is that the model Eq. (1) is epidemiologically meaningful, as it proved that all the state variables are non-negative and bounded. That is, no solution path can leave through any boundary of $\Theta$ and it is sufficient to consider the dynamics of the model (1) in $\Theta$. In this region, the model is considered to be mathematically and epidemiologically well posed.

### Analysis of the sub models

#### COVID-19 sub-model

Setting $I_H=0$, $I_A=0$, $I_{HC}=0$, $I_{AC}=0$, $D_{HC}=0$ for (1), we obtain the COVID-19 sub-model which is given by:

2 $$\begin{aligned} {\left\{ \begin{array}{ll} &{}S^\prime = (1-\rho ) \Lambda + \psi R - \left( D_1 + \lambda _C \right) S,\\ &{}V^\prime = \rho \Lambda + \xi S - \left( \mu + \sigma \lambda _C\right) V, \\ &{}I_C^\prime = \lambda _C S + \sigma \lambda _C V - D_2 I_C, \\ &{}R^\prime = \theta I_C - D_3 R, \end{array}\right. }, \end{aligned}$$

where the force of infection of the COVID-19 sub model (1) is $\lambda _C = \gamma _c \frac{I_C}{N}$.

#### Equilibria and basic reproductive number

Setting $I_C^\prime = 0$ in (2), we obtain the COVID-19 free equilibrium which is denoted by $E_{c}^0$. It follows that,

$$\begin{aligned} E_{c}^0=\left( \frac{\Lambda (1-\rho )}{D_1}, \frac{\Lambda (\rho \mu +\xi )}{\mu D_1}, \ 0, \ 0\right) . \end{aligned}$$

Implementing the theorem in Van den Driessche and Watmough^36^, the basic reproduction number of COVID-19 sub-model, denoted by ${\mathscr {R}}_{0c}$, is given by

$$\begin{aligned} {\mathscr {R}}_{0c}=\frac{ (1-\rho )\mu \gamma _c}{D_1 D_2} + \frac{\sigma (\rho \mu + \xi ) \gamma _c}{D_1 D_2}. \end{aligned}$$

The reproductive number tells us how many secondary cases will one infected individual produce in an entirely susceptible population of hosts. In other words, it tells the number of infected people that are generated by the introduction of a single infected person into a susceptible population. The epidemiological interpretations of each term in ${\mathscr {R}}_{0c}$ are presented in the following manner:

- The first term in ${\mathscr {R}}_{0c}$, given by $\frac{ (1-\rho )\mu \gamma _c}{D_1 D_2}$, gives the number of secondary infections of susceptible individuals that one COVID-19 infected individual can produce in a disease-free population.
- The second term in ${\mathscr {R}}_{0c}$, given by $\frac{\sigma (\rho \mu + \xi ) \gamma _c}{D_1 D_2}$, gives the number of secondary infections of vaccinated individuals that one COVID-19 infected individual can produce in a disease-free population.

In the absence of vaccination, when $\sigma =0$ and $\xi = 0$, ${\mathscr {R}}_{0c}$ is given by

$$\begin{aligned} {\mathscr {R}}_{0c}^*= {\mathscr {R}}_{0c}=\frac{\gamma _c}{D_2}. \end{aligned}$$

Biologically, ${\mathscr {R}}_{0c}^*$ represents the number of secondary infections caused by one infected individual during the mean course of infection $\left( {1}/{D_2}\right)$ in a completely susceptible population.

The controlled reproduction number, ${\mathscr {R}}_{0c}$ can be written as

$$\begin{aligned} {\mathscr {R}}_{0c}=\left( \frac{(1-\rho )\mu + \sigma (\rho \mu + \xi )}{D_1}\right) {\mathscr {R}}_{0c}^*. \end{aligned}$$

Which implies,

$$\begin{aligned} \frac{\partial {\mathscr {R}}_{0c}}{\partial \xi } = -\frac{ (1-\rho ) (1-\sigma ) \mu }{D_1}{\mathscr {R}}_{0c}^*<0, \ \ \ \lim \limits _{\xi \rightarrow \infty }{\mathscr {R}}_{0c} = \sigma {\mathscr {R}}_{0c}^*\ \ \ \ \text {and} \ \ \ \ {\mathscr {R}}_{0c}<{\mathscr {R}}_{0c}^*. \end{aligned}$$

That is,

$$\begin{aligned} \sigma {\mathscr {R}}_{0c}^*< {\mathscr {R}}_{0c} < {\mathscr {R}}_{0c}^*. \end{aligned}$$

If ${\mathscr {R}}_{0c}^*<1$, then ${\mathscr {R}}_{0c} < 1$. This show that vaccination coverage plays an important role in controlling COVID-19 disease. However, for ${\mathscr {R}}_{0c}^*> 1$

$$\begin{aligned} \sigma {\mathscr {R}}_{0c}^*<1 \ \ \ \text {if and only if} \ \ \ \sigma > \frac{1}{{\mathscr {R}}_{0c}^*}. \end{aligned}$$

Thus, when ${\mathscr {R}}_{0c}^*>1$ elimination of the COVID-19 from the population relies on the vaccine efficacy, not on the vaccine coverage. For ${\mathscr {R}}_{0c} = 1$, i.e., for $\sigma {\mathscr {R}}_{0c}^*<1$, there exists a critical vaccination proportion that will achieve eradication. Thus, the critical vaccination proportion for eradication of the disease, $\xi ^C$, is given by

$$\begin{aligned} \xi ^C = \frac{\mu (1-{\mathscr {R}}_{0c}^*)}{\sigma {\mathscr {R}}_{0c}^*- 1}. \end{aligned}$$

##### Theorem 4.2

The COVID-19-free equilibrium $E_c^0$ is locally asymptotically stable whenever ${\mathscr {R}}_{0c} < 1$ and unstable if ${\mathscr {R}}_{0c} > 1$.

##### Proof

Jacobian of (2) at $E_c^0$, denoted by ${\mathscr {J}}$, is

$$\begin{aligned} {\mathscr {J}} = \begin{pmatrix} -D_1 &{} 0 &{} -\frac{\mu (1-\rho ) \gamma _c}{D_1} &{} \psi \\ \xi &{} -\mu &{} -\frac{\sigma \gamma _c (\mu \rho +\xi )}{D_1} &{} 0 \\ 0 &{} 0 &{} -D_2 (1-{\mathscr {R}}_{0c}) &{} 0 \\ 0 &{} 0 &{} \theta &{} -D_3 \end{pmatrix}. \end{aligned}$$

The eigenvalues of ${\mathscr {J}}$ are: $-\mu$, $-D_1$ and $-D_2 (1-{\mathscr {R}}_{0c})$. We can see that all eigenvalues are negative provided that ${\mathscr {R}}_{0c}<1$. Therefore, we can conclude that $E_c^0$ is locally asymptotically stable whenever ${\mathscr {R}}_{0c}<1$. $\square$

The epidemiological implication of *Theorem* 4.2 is that the control of the transmission of COVID-19 can be accomplished by setting parameter values so that ${\mathscr {R}}_{0c}<1$ if the initial size of the subpopulations involved in (2) are in the basin of attraction of $E_c^0$. Thus, a sufficiently small flow of infectious individuals will not generate an outbreak, and the disease can be eliminated from the community. On the other hand, if ${\mathscr {R}}_{0c}>1$, the entire population will get infected quickly because a single infected person can spread COVID-19 to several people.

The endemic equilibrium point is represented by $E_c^*= (S^*, V^*, I_C^*, R^*)$ and it is obtained by equating system (2) to zero and solving for state variables. Indeed, we come up with the following equilibrium points,

3 $$\begin{aligned} S^*= \frac{(1-\rho ) \Lambda + \psi R^*}{D_1 + \lambda _C^*}, \ \ V^*= \frac{\rho \Lambda + \xi S^*}{\mu + \sigma \lambda _C^*}, \ \ I_C^*= \frac{(S^*+ \sigma V^*) \lambda _C^*}{D_2} \ \text {and} \ R^*= \frac{\theta I_C^*}{D_3}. \end{aligned}$$

Substituting (3) into $\lambda _C^*$ implies that the endemic equilibrium of the model (2) meets the following equation.

$$\begin{aligned} \lambda _C^*\left( K \lambda _C^{*^2} + L \lambda _C^*+ M\right) = 0, \end{aligned}$$

where

$$\begin{aligned} K= & {} \sigma \left( \theta + D_3\right) , \\ L= & {} \frac{D_1 D_2 {\mathscr {R}}_{0c} \left( \theta + D_3\right) }{\gamma _c} +\rho (1 - \sigma ) \left( D_2 D_3 - \theta \psi \right) + \sigma D_2 D_3 -\sigma \gamma _c D_3\ \ \text {and}\\ M= & {} D_1 D_2 D_3 \left( 1 - {\mathscr {R}}_{0c}\right) . \end{aligned}$$

The COVID-19-free equilibrium $E_c^0$ is obtained for $\lambda _C^*=0$. The endemic equilibrium corresponds to the solution of

4 $$\begin{aligned} K \lambda _C^{*^2} + L \lambda _C^*+ M = 0. \end{aligned}$$

The endemic equilibrium is obtained by substituting the solution of (4) in $S^*$, $V^*$, $I^*$ and $R^*$. If ${\mathscr {R}}_{0c}>1$(or $M<0$), then (4) has a unique positive root $\lambda _C^*=\frac{-L+\sqrt{\Delta }}{2K}$, where $\Delta =L^2-4KM>0$. If ${\mathscr {R}}_0=1$(or $M=0$), then (4) has a unique positive solution $\lambda _C^*=\frac{-L}{2K}$, provided that $L<0$. Here, if $L=0$, then $\lambda _C^*=0$, which in turn gives $E_c^0$, and if $L>0$, then $\lambda _C^*<0$ and this does not make sense in epidemiology. For ${\mathscr {R}}_{0c}<1$(or $M>0$) and $\Delta >0$, we consider two cases:

- Case1: If $L>0$, then $\lambda _C^*<0$, that is (4) had no positive solution.
- Case2: If $L<0$, that is if $L<-2\sqrt{KM}<0$, then (4) had two endemic equilibria.

Considering these different cases for the solution of (4), a theorem is established as follows.

##### Theorem 4.3

The sub-model (2) has

1. A unique endemic equilibrium if
  - $M < 0$ if and only if ${\mathscr {R}}_{0c} > 1$;
  - $$L < 0$$ and $$M = 0$$ or $$L^2 - 4KM = 0$$;
2. Two endemic equilibria if $M > 0, \ L < 0$ and $L^2 - 4KM > 0$;
3. No endemic equilibrium otherwise.

When the ${\mathscr {R}}_{0c}<1$, the second case of Theorem 4.3 suggests the probability of backward bifurcation where a stable COVID-19-free and a stable endemic equilibrium coexist.

#### Backward bifurcation analysis

Backward bifurcation analysis of COVID-19 sub-model (2) was carried out by employing the center manifold theory^37^. We modify variables in the following manner to implement the center manifold theory.

$$\begin{aligned} x_1 = S, \ x_2 = V,\ x_3 = I_C, \ x_4 = R, \end{aligned}$$

so that,

$$\begin{aligned} N = x_1 +x_2+x_3+x_4 \ \ \text {and} \ \ \lambda _C=\frac{\gamma _c x_3}{N}. \end{aligned}$$

Moreover, with $x = \left( x_1, \ x_2, \ x_3,\ x_4\right) ^T$, system (2) can be expressed as

$$\begin{aligned} x^\prime = \left( f_1, \ f_2, \ f_3, \ f_4\right) ^T. \end{aligned}$$

That is also,

5 $$\begin{aligned} {\left\{ \begin{array}{ll} \frac{dx_1}{dt} = f_1 = (1-\rho ) \Lambda + \psi x_4 - \left( D_1 + \lambda _C \right) x_1,\\ \frac{dx_2}{dt} = f_2 = \rho \Lambda + \xi x_1 - \left( \mu + \sigma \lambda _C\right) x_2, \\ \frac{dx_3}{dt} = f_3 = \lambda _C x_1 + \sigma \lambda _C x_2 - D_2 x_3,\\ \frac{dx_4}{dt} = f_4 = \theta x_3 - D_3 x_4. \end{array}\right. } \end{aligned}$$

The Jacobian of the system (5) at $E_c^0$ is given by,

6 $$\begin{aligned} \begin{pmatrix} -D_1 &{} 0 &{} -\frac{\gamma _c \mu (1-\rho )}{D_1} &{} \psi \\ \xi &{} -\mu &{} -\frac{\gamma _c \sigma (\mu \rho +\xi )}{D_1} &{} 0 \\ 0 &{} 0 &{} \frac{\gamma _c ((1-\rho )\mu + \sigma (\rho \mu + \xi ))}{D_1}-D_2 &{} 0 \\ 0 &{} 0 &{} \theta &{} -D_3 \\ \end{pmatrix}. \end{aligned}$$

Choosing $\gamma _c$ as the bifurcation parameter and setting ${\mathscr {R}}_{0c} = 1$ gives

$$\begin{aligned} \gamma _c^*= \gamma _c =\frac{D_1 D_2 }{((1-\rho )\mu + \sigma (\rho \mu + \xi ))}. \end{aligned}$$

For $\gamma _c = \gamma _c^*$, equation (6) has eigenvalues $-\mu -\xi$, $-\mu$, $-D_3$ and 0. We, therefore, apply the Center Manifold Theorem to analyze the dynamics of (5) about $\gamma _c = \gamma _c^*$.

The right-eigenvector ${\textbf{w}}=(w_1, \ w_2, \ w_3, \ w_4)^T$ related to zero eigenvalue of (6) is given by

$$\begin{aligned} w_4&=\frac{\theta w_3}{D_3}, \ \ w_1 = \frac{1}{D_1 }\left( w_4 \psi -\frac{\gamma _c \mu (1-\rho ) w_3}{D_1 }\right) \\ w_2&=\frac{1}{\mu }\left( \xi w_1-\frac{\gamma _c \sigma w_3 (\mu \rho +\xi )}{D_1}\right) , \ \ w_3 > 0. \end{aligned}$$

Similarly, the left-eigenvector ${\textbf{u}}=(u_1, \ u_2, \ u_3, \ u_4)^T$ associated with the zero eigenvalue is given by

$$\begin{aligned} u_1 = 0, \ u_2 = 0, \ u_3 > 0, \ u_4 = 0. \end{aligned}$$

There is no need to calculate the derivatives of $f_1$, $f_2$ and $f_4$. They are zero since $u_1$, $u_2$ and $u_3$ are all zero. From the derivatives of $f_3$, coefficients *a* and *b* defined in^37^ are computed as follows:

$$\begin{aligned} a= & {} \sum _{k, i, j=1}^{4}v_kw_iw_j\frac{\partial ^2 f_k }{\partial x_i \partial x_j}\left( E_c^0, \gamma _c^*\right) \\= & {} \sum _{i, j=1}^{4}\left[ v_3w_iw_j\frac{\partial ^2 f_3 }{\partial x_i \partial x_j}\left( E_c^0, \gamma _c^*\right) \right] \\= & {} \frac{2 \gamma _c^*\mu v_3 w_3^2}{D_1 D_3 \Lambda }\left[ \sigma \gamma _c^*D_3-\left( \rho (1 - \sigma ) \left( D_2 D_3 - \theta \psi \right) + \sigma D_2 D_3 + \frac{D_1 D_2 \left( \theta + D_3\right) }{\gamma _c^*}\right) \right] \\ \text {and}\\ b= & {} \sum _{k,j=1}^{4}v_kw_j\frac{\partial ^2 f_k }{\partial x_j \partial \beta }\left( E_c^0, \gamma _c^*\right) = \sum _{i=1}^{4}v_3w_i\frac{\partial ^2 f_3 }{\partial x_i \partial \beta }\left( E_c^0, \gamma _c^*\right) =\frac{D_2 v_3 w_3}{\gamma _c^*} \end{aligned}$$

**Figure 2:**
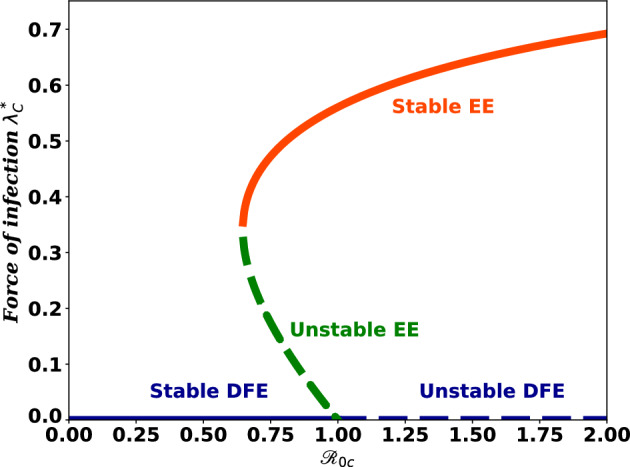
Backward bifurcation diagram for COVID-19 sub-model.

From this result, we can observe that the COVID-19 sub-model exhibits backward bifurcation whenever $a>0$. If the vaccine is perfect and there is no reinfection possibility for vaccinated individuals, that is, if $\sigma = 0$, the value of *a* becomes negative, which implies system (2) has a trans-critical bifurcation. The epidemiological significance of backward bifurcation is that, besides making ${\mathscr {R}}_{oc}<1$, it needs more action to reduce COVID-19 disease transmission through the population. Figure 2 show the phenomenon of backward bifurcation as evidence of the COVID-19 sub-model analysis. A stable equilibrium is represented by a solid line and an unstable one by a dashed line. It confirms the analytical result, which show that endemic equilibrium exists when ${\mathscr {R}}_{oc}<1$.

### HIV/AIDS sub-model

The HIV/AIDS sub-model is given by:

7 $$\begin{aligned} {\left\{ \begin{array}{ll} &{}S^\prime = (1-\rho ) \Lambda - \left( D_1+\lambda _H \right) S, \\ &{}V^\prime = \rho \Lambda + \xi S - \left( \mu +\lambda _H \right) V,\\ &{}I_H^\prime = (S + V)\lambda _H - D_4 I_H,\\ &{}I_A^\prime = \omega I_H - D_5 I_A, \end{array}\right. } \end{aligned}$$

where $$\lambda _H = \gamma _h \frac{I_H+\epsilon I_A}{N}$$.

If $I_H^\prime =I_A^\prime = 0$, we obtain the HIV/AIDS-free equilibrium, denoted by $E_{h}^0$, which is given by,

$$\begin{aligned} E_{h}^0=\left( \frac{\Lambda (1-\rho )}{D_1}, \frac{\Lambda (\rho \mu +\xi )}{\mu D_1}, \ 0, \ 0\right) . \end{aligned}$$

The reproduction number for the HIV/AIDS sub-model was established by using the next-generation matrix method on system (7). The disease (infected) compartments of (7) are $I_H$ and $I_A$ and the non-disease (noninfected) compartments are S and V. Taking the infected compartments with their dynamics we have,

$$\begin{aligned} {\mathscr {F}} = \begin{pmatrix} (S+V) \lambda _H \\ 0 \end{pmatrix}\ \ \ \ \text {and} \ \ \ \ {\mathscr {V}}=\begin{pmatrix} D_4 I_H \\ D_5 I_A-\omega I_H \end{pmatrix}. \end{aligned}$$

The Jacobian matrix of ${\mathscr {F}}$ and ${\mathscr {V}}$ , denoted by *F* and *V*, at $E_{h}^0$ is given by

$$\begin{aligned} F = \begin{pmatrix} \gamma _h &{} \epsilon \gamma _h \\ 0 &{} 0 \end{pmatrix} \ \ \ \ \text {and} \ \ \ \ V=\begin{pmatrix} D_4 &{} 0 \\ -\omega &{} D_5 \end{pmatrix}. \end{aligned}$$

Hence, we have

$$\begin{aligned} FV^{-1} = \begin{pmatrix} \frac{\gamma _h }{D_4}\left( 1+\frac{\omega \epsilon }{D_5} \right) &{} \frac{\epsilon \gamma _h}{D_5} \\ 0 &{} 0 \end{pmatrix}. \end{aligned}$$

The corresponding eigenvalues are $\frac{\gamma _h }{D_4}\left( 1+\frac{\omega \epsilon }{D_5} \right)$ and 0. The basic reproduction number of HIV/AIDS sub-model, denoted by ${\mathscr {R}}_{0h}$, is given by

$$\begin{aligned} {\mathscr {R}}_{0h}=\frac{\gamma _h }{D_4}\left( 1+\frac{\omega \epsilon }{D_5} \right) . \end{aligned}$$

#### Theorem 4.4

The HIV/AIDS-free equilibrium $E_{h}^0$ is locally asymptotically stable whenever ${\mathscr {R}}_{0h} < 1$ and unstable if ${\mathscr {R}}_{0h} > 1$.

#### Proof

The Jacobian at the disease-free equilibrium is

$$\begin{aligned} \begin{pmatrix} -D_1 &{} 0 &{} \frac{\mu (\rho -1) \gamma _h}{D_1} &{} \frac{\mu (\rho -1) \epsilon \gamma _h}{D_1} \\ \xi &{} -\mu &{} -\frac{\gamma _h (\mu \rho +\xi )}{D_1 } &{} -\frac{\epsilon \gamma _h (\mu \rho +\xi )}{D_1} \\ 0 &{} 0 &{} \gamma _h-D_4 &{} \epsilon \gamma _h \\ 0 &{} 0 &{} \omega &{} -D_5 \end{pmatrix}. \end{aligned}$$

It is easy to see that $-\mu$ and $-D_1$ are negative eigenvalues. The remaining two eigenvalues are the eigenvalues of the $2 \times 2$ matrix

$$\begin{aligned} \begin{pmatrix} \gamma _h-D_4 &{} \epsilon \gamma _h \\ \omega &{} -D_5 \end{pmatrix}. \end{aligned}$$

The characteristic equation takes the form

8 $$\begin{aligned} \ell ^2 + a_1 \ell + a_2 = 0 \, \end{aligned}$$

where $$a_1 = \left( D_5 +D_4 \left( 1-\frac{\gamma _h}{D_4}\right) \right)$$ and $$a_2 = D_4 D_5 \left( 1-{\mathscr {R}}_{0h}\right)$$.

The stability of $E_{h}^0$ relies on the sign of the roots of Eq. (8). By Routh-Hurwitz condition^38^, Eq. (8) has negative real part if $a_1>0$ and $a_2>0$. For ${\mathscr {R}}_{0h} < 1$, it is easy to see that the condition $a_1, a_2 > 0$ are satisfied. Therefore, $E_{h}^0$ is locally asymptotically stable whenever ${\mathscr {R}}_{0h} < 1$. If ${\mathscr {R}}_{0h} > 1$, $a_2 >0$ and Descartes rule of sign^39^ implies (8) has a real positive solution. Which implies the disease-free equilibrium $E_{h}^0$ is unstable for ${\mathscr {R}}_{0h} > 1$. $\square$

The biological implication of *Theorem* 4.4 is that a sufficiently small flow of HIV/AIDS infected individuals will not generate an outbreak of the disease unless ${\mathscr {R}}_{0h} > 1$.

#### Theorem 4.5

The HIV/AIDS-free equilibrium $E_{h}^0$ is globally asymptotically stable for ${\mathscr {R}}_{0h} < 1$.

#### Proof

Consider the Lyapunov function given by,

$$\begin{aligned} L=\frac{1}{D_4}\left( 1+\frac{\omega \epsilon }{D_5} \right) I_H + \frac{\epsilon }{D_5} A. \end{aligned}$$

$L=0$ for $I_H, \ I_A = 0$ and $L>0$ for $I_H, \ I_A \ne 0$. Furthermore,

$$\begin{aligned} L^\prime= & {} \frac{1}{D_4}\left( 1+\frac{\omega \epsilon }{D_5} \right) I_H^\prime + \frac{\epsilon }{D_5} A^\prime \\= & {} \frac{1}{D_4}\left( 1+\frac{\omega \epsilon }{D_5} \right) \left( \frac{(S+V)}{N}\gamma _h \left( I_H + \epsilon I_A\right) - D_4 I_H \right) + \frac{\epsilon }{D_5}\left( \omega I_H - D_5 I_A\right) \\\le & {} \left( {\mathscr {R}}_{0h}-1\right) \left( I_H +\epsilon I_A\right) \end{aligned}$$

Thus, $L^\prime < 0$ for ${\mathscr {R}}_{0h}<1$ and $L^\prime = 0$ for ${\mathscr {R}}_{0h} = 1$ or $I_H=0$. It follows that, if $I_H=0$, then $S \rightarrow \frac{\Lambda (1-\rho )}{D_1}$ and $V\rightarrow \frac{\Lambda (\rho \mu +\xi )}{\mu D_1}$ as $t \rightarrow \infty$. That is, $\{E_h^0\}$ is the largest invariant set such that $L^\prime =0$. Following^40^, we can conclude that $\{E_h^0\}$ is globally asymptotically stable. That is, when ${\mathscr {R}}_{0h} < 1$, all solutions of (7) converge to the disease-free equilibrium $E_h^0$. $\square$

The epidemiological implication of this *Theorem *4.5 is that independent of the initially available sub-populations in model (7), HIV/AIDS infected individuals will not lead to the large outbreaks and disease will die out in the long run. That is, the HIV/AIDS infected population vanishes in time.

#### Theorem 4.6

The HIV/AIDS sub-model has a unique endemic equilibrium if and only if ${\mathscr {R}}_{0h} > 1$.

#### Proof

The endemic equilibrium of HIV/AIDS sub-model, $E_H^*= \left( S^*, V^*, I_H^*, I_A^*\right)$, is given by

9 $$\begin{aligned} S^*= \frac{(1-\rho )\Lambda }{D_1 + \lambda _H^*}, \ V^*= \frac{\rho \Lambda +\xi S^*}{\mu + \lambda _H^*}, \ I_A^*=\frac{\omega }{D_5}I_H^*,\ I_H^*= \frac{\left( S^*+ V^*\right) \lambda _H^*}{D_4} \end{aligned}$$

Substituting (9) into $\lambda _H^*$, we obtain

10 $$\begin{aligned} \lambda _H^*\left( \lambda _H^*-\frac{D_4D_5\left( {\mathscr {R}}_{0h}-1\right) }{\omega + D_5} \right) =0. \end{aligned}$$

From (10), $\lambda _H^*= \frac{D_4D_5({\mathscr {R}}_{0h}-1)}{\omega + D_5}$ is used to establish the endemic equilibrium $E_H^*$. It is clear that $\lambda _H^*> 0$ if and only if ${\mathscr {R}}_{0h} > 1$. Thus, HIV/AIDS sub-model (7) has a unique positive solution whenever ${\mathscr {R}}_{0h} > 1$. Furthermore, ${\mathscr {R}}_{0h} < 1$ implies that the force of infection $\lambda _H^*$ is negative (which is biologically meaningless). In this instance, the model does not have a positive equilibrium. Epidemiologically, this result implies that once the disease invades the population, the epidemic persists, and the number of population in *S*, *V*, $I_H$ and $I_A$ eventually approach the numbers $S^{*}$, $V^{*}$, $I_H^{*}$ and $I_A^{*}$, respectively. $\square$

### Analysis of the co-infection model

COVID-19 and HIV/AIDS co-infection model has a disease-free equilibrium, denoted by $E^0$, given by

$$\begin{aligned} E^0=\left( \frac{\Lambda (1-\rho )}{D_1}, \frac{\Lambda (\rho \mu +\xi )}{\mu D_1},\ 0,\ 0,\ 0,\ 0,\ 0,\ 0,\ 0\right) . \end{aligned}$$

The next generation matrix approach, established in^36^, is used to determine the reproduction number as follows. The rate of appearance of new infection and rate of transfer from one compartment to another in the infectious classes gives the following:

$$\begin{aligned} {\mathscr {F}}=\begin{pmatrix} (S+\sigma V)\lambda _C\\ (S+V)\lambda _H \\ 0 \\ \lambda _C I_H +\lambda _H I_C\\ \kappa \lambda _C \\ 0 \end{pmatrix} \ \text {and}\ {\mathscr {V}} = \begin{pmatrix} \left( D_2 + \lambda _H\right) I_C \\ \left( D_4 + \lambda _C\right) I_H-\alpha I_{HC} \\ \left( \kappa \hbox { }\ \lambda _C+ D_5\right) I_A +\omega I_{H}+ \varphi I_{HC} \\ D_6 I_{HC_A} \\ D_7 I_{AC}-\phi I_{HC} \\ D_8 D_{HC}-\eta _1 I_{HC}-\eta _2 I_{AC} \end{pmatrix}. \end{aligned}$$

The Jacobian of ${\mathscr {F}}$ and ${\mathscr {V}}$ at disease-free equilibrium $E^0$ are given as follows,

$$\begin{aligned} F= & {} \begin{pmatrix} \frac{\gamma _c ((1-\rho ) \mu +\sigma (\mu \rho +\xi ))}{D_1} &{} 0 &{} 0 &{} \frac{\gamma _c ((1-\rho ) \mu + \sigma (\mu \rho +\xi )+)}{D_1} &{} \frac{\gamma _c ((1-\rho )\mu + \sigma (\mu \rho +\xi ))}{D_1} &{} 0 \\ 0 &{} \gamma _h &{} \epsilon \gamma _h &{} \gamma _h &{} \epsilon \gamma _h &{} 0 \\ 0 &{} 0 &{} 0 &{} 0 &{} 0 &{} 0 \\ 0 &{} 0 &{} 0 &{} 0 &{} 0 &{} 0 \\ 0 &{} 0 &{} 0 &{} 0 &{} 0 &{} 0 \\ 0 &{} 0 &{} 0 &{} 0 &{} 0 &{} 0 \end{pmatrix} \ \text {and} \\ V= & {} \begin{pmatrix} D_2 &{} 0 &{} 0 &{} 0 &{} 0 &{} 0 \\ 0 &{} D_4 &{} 0 &{} -\alpha &{} 0 &{} 0 \\ 0 &{} -\omega &{} D_5 &{} 0 &{} -\varphi &{} 0 \\ 0 &{} 0 &{} 0 &{} D_6 &{} 0 &{} 0 \\ 0 &{} 0 &{} 0 &{} -\phi &{} D_7 &{} 0 \\ 0 &{} 0 &{} 0 &{} -\eta _1 &{} -\eta _2 &{} D_8 \end{pmatrix}. \end{aligned}$$

The basic reproduction number, ${\mathscr {R}}_0$, for the COVID-19-HIV/AIDS co-infection model is the maximum of eigenvalues of the next generation matrix, $FV^{-1}$. That is,

$$\begin{aligned} {\mathscr {R}}_0 =max\left\{ 0,\ \frac{\gamma _c ((1-\rho )\mu + \sigma (\rho \mu + \xi ))}{D_1 D_2},\ \frac{\gamma _h }{D_4}\left( 1+\frac{\omega \epsilon }{D_5} \right) \right\} . \end{aligned}$$

Thus,

$$\begin{aligned} {\mathscr {R}}_0=max\left\{ {\mathscr {R}}_{0c},\ {\mathscr {R}}_{0h}\right\} . \end{aligned}$$

This implies that the dynamics of COVID-19 and HIV/AIDS co-infection will be dominated by the disease with the bigger basic reproduction number. From Theorem 2 of^36^, the following theorem is deduced.

#### Theorem 4.7

The disease-free equilibrium $E^0$ is locally asymptotically stable if ${\mathscr {R}}_0<1$ and unstable if ${\mathscr {R}}_0>1$.

To investigate the global stability of $E^0$ we apply the approach in^41^. First, we write system (1) in the form

$$\begin{aligned} \frac{d{\textbf{X}}}{dt}= & {} F({\textbf{X}},{\textbf{I}})\\ \frac{d{\textbf{I}}}{dt}= & {} G({\textbf{X}},{\textbf{I}}), \ G({\textbf{X}}, {\textbf{0}}) = {\textbf{0}} \end{aligned}$$

where ${\textbf{X}}=(S, V, R)$ and ${\textbf{I}}= (I_C, I_H, I_A, I_{HC}, I_{AC}, D_{HC})$ denotes uninfected and infected population respectively. $U_0 = ({\textbf{X}}^*, {\textbf{0}})$ denotes the disease-free equilibrium of system (1). Furthermore, suppose

**H1**: For $\frac{d{\textbf{X}}}{dt}= F({\textbf{X}}, {\textbf{0}})$, $\mathbf {X^*}$ is globally asymptotically stable,
**H2**: $G({\textbf{X}}, {\textbf{I}}) = A{\textbf{I}} - {\hat{G}}({\textbf{X}}, {\textbf{I}}),\ {\hat{G}}({\textbf{X}},{\textbf{I}}) \ge 0$ for $({\textbf{X}},{\textbf{I}}) \in \Theta$, where $A = D_{\textbf{I}} G(\mathbf {X^*}, {\textbf{0}})$ is an M-matrix (the off-diagonal elements of A are nonnegative).

#### Theorem 4.8

The disease-free equilibrium $U_0 = ({\textbf{X}}^*, {\textbf{0}})$ is a globally asymptotic stable equilibrium of (1) provided that ${\mathscr {R}}_0<1$ and that assumptions (Hl) and (H2) are satisfied.

#### Proof

Writing system (1) for investigating H1 and H2, we have

$$\begin{aligned} F({\textbf{X}}, {\textbf{0}})=\begin{pmatrix} (1-\rho ) \Lambda + \psi R - D_1 S\\ \rho \Lambda + \xi S - \mu V\\ -D_3 R\\ \end{pmatrix}, \\ A= \begin{pmatrix} -D_2 \left( 1-{\mathscr {R}}_0\right) &{} 0 &{} 0 &{} D_2 {\mathscr {R}}_0 &{} D_2 {\mathscr {R}}_0 &{} 0 \\ 0 &{} \gamma _h-D_4 &{} \epsilon \gamma _h &{} \alpha +\gamma _h &{} \epsilon \gamma _h &{} 0 \\ 0 &{} \omega &{} -D_5 &{} 0 &{} \varphi &{} 0 \\ 0 &{} 0 &{} 0 &{} -D_6 &{} 0 &{} 0 \\ 0 &{} 0 &{} 0 &{} \phi &{} -D_7 &{} 0 \\ 0 &{} 0 &{} 0 &{} \eta _1 &{} \eta _2 &{} -D_8 \end{pmatrix} \end{aligned}$$

and

$$\begin{aligned} {\hat{G}}({\textbf{X}},{\textbf{I}})= \begin{pmatrix} \left( D_2 {\mathscr {R}}_{oc}-\frac{(S+\sigma V) \gamma _c}{N} \right) \left( I_C+I_{HC}+I_{AC}\right) + \lambda _H I_C\\ \gamma _h\left( 1-\frac{S+V}{N}\right) \left( I_H+I_{HC}+I_{AC}\right) + \lambda _C I_H\\ \kappa \lambda _C I_A\\ -\lambda _C I_H-\lambda _H I_C\\ -\kappa \lambda _C I_A\\ 0 \end{pmatrix}. \end{aligned}$$

The matrix *A* is M-matrix, but all rows of ${\hat{G}}({\textbf{X}},{\textbf{I}})$ are not non negative for all $({\textbf{X}},{\textbf{I}}) \in \Theta$. So assumption H2 is not satisfied. Hence, the disease-free equilibrium $E^0$ may not be globally stable, and backward bifurcation may occur in the system (1). That is, the co-infection may persist even if the dominant reproduction number (${\mathscr {R}}_0$) is less than unity. However, if maximum protection is provided against the COVID-19 and HIV/AIDS co-infection, that is, if the third and fourth rows of ${\hat{G}}({\textbf{X}},{\textbf{I}})$ are positive, then global stability of disease free equilibrium may be achieved. That is to say, if incidences of co-infection are kept to an absolute minimum, efforts made to fight COVID-19 and HIV/AIDS may become successful. $\square$

### Effects of control parameters and transmission rate on ${\mathscr {R}}_{0c}$ and ${\mathscr {R}}_{0h}$

In this section, the impact of some parameters on reproduction numbers is analyzed qualitatively. Effects of the control parameter, $\xi$ and $\theta$, and transmission rates, $\gamma _c$, analysis are performed by evaluating the partial derivatives of the basic reproduction numbers with respect to these parameters. We can see that

$$\begin{aligned} \frac{\partial {\mathscr {R}}_{0c}}{\partial \xi }=-\frac{\mu (1-\rho ) (1-\sigma ) \gamma _c}{D_1^2 D_2}< 0,{} & {} \frac{\partial {\mathscr {R}}_{0c}}{\partial \theta }=-\frac{\gamma _c ((1-\rho )\mu + \sigma (\mu \rho +\xi ))}{D_1 D_2^2}< 0,\\ \frac{\partial {\mathscr {R}}_{0c}}{\partial \gamma _c} =\frac{((1-\rho )\mu + \sigma (\rho \mu + \xi ))}{D_1 D_2}>0,{} & {} \frac{\partial {\mathscr {R}}_{0h}}{\partial \gamma _h} = \frac{1}{D_4}\left( 1+\frac{\omega \epsilon }{D_5} \right) >0. \end{aligned}$$

**Figure 3:**
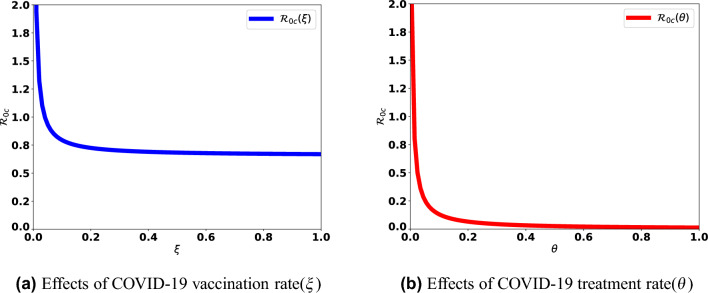
${\mathscr {R}}_{0c}$ for different values of vaccination and treatment rates.

The reproduction number ${\mathscr {R}}_{0c}$ is a decreasing function with respect to the control parameters, $\xi$ and $\theta$. That means, for every value of $\xi$ and $\theta$, the vaccination of susceptible individuals and treating COVID-19-infected individuals step-down ${\mathscr {R}}_{0c}$ (see Fig. 3) and therefore reduce the COVID-19 and HIV/AIDS co-infection cases. On the other hand, ${\mathscr {R}}_{0c}(\gamma _c)$ and ${\mathscr {R}}_{0h}(\gamma _h)$ are increasing functions for any $\gamma _c$ and $\gamma _h$, respectively.

### Parametric estimation

Ethiopia is considered in estimating some model parameters. The scarcity of data on HIV and COVID-19 co-infection makes the fitting on real COVID-19 data only. The total number of people who have contracted COVID-19 each month since March 2020, when the disease first appeared in Ethiopia, was compiled from^42^. Table 2 displays this data. To fit the model to the actual data, the Python library’s scipy function scipy.optimize.curve_fit(), an algorithm based on a Levenburg-Marquardt method to fit functions to data, is employed. Indeed, the required Python libraries were imported and the function that is to be fitted to the existing data was defined. That is, system (1) with initial values was written as

11 $$\begin{aligned} \mathbf {x^\prime } = g(t, {\textbf{x}}, {\textbf{p}}), \ \ \mathbf {x(t_0)} = \mathbf {\mathbf {x_0}}, \end{aligned}$$

where ${\textbf{p}}$ is a vector of parameters for the model that need to be found and ${\textbf{x}}$ is the vector of state variables.

The aim of the parametric estimation was to determine the parameter vector ${\textbf{p}}$ so that the sum of the square of residuals

12 $$\begin{aligned} \sum _{i=1}^{n}\left( \mathbf {x_i}-\varvec{{\tilde{x}}_i}\right) ^2 \end{aligned}$$

is as small as possible, where $\varvec{{\tilde{x}}_i}$ is the real data and $\mathbf {x_i} = {\textbf{x}}(t_i, {\textbf{p}})$ is a solution to (11) at time $t_i$ for a given ${\textbf{p}}$. In this regard, the curve_fit function returns the tuple of optimal parameters, in the sense that minimizing Eq. (12) subject to (11). The details of procedures for using scipy in fitting data are given in^43^.

**Figure 4:**
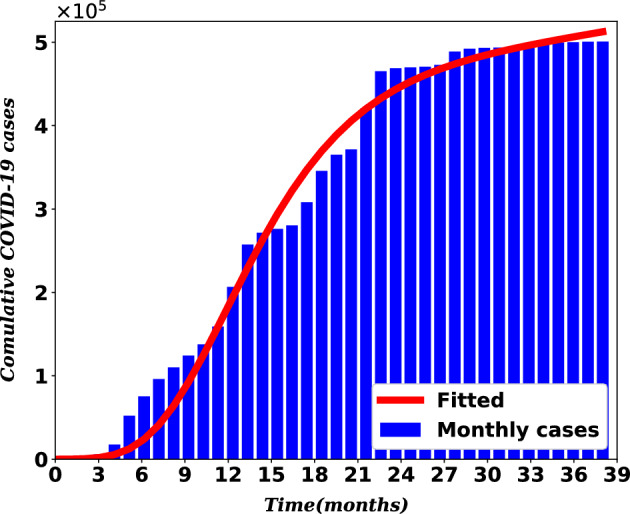
Model fit to real data.

Data for the total population and the life expectancy of Ethiopia for the year 2021 were obtained from^44^. The total population and life expectancy are estimated to be 120, 283, 026 and 65 years, respectively. It follows that $\mu =\frac{1}{(65 \times 12)}$ per month and $\Lambda =\mu \times N = 154209$ people per month. On March 31, 2020, 25 people were found to be COVID-19 infected and 2 people were recovered from COVID-19. Since no vaccine was available and no co-infection was reported in the first month of the outbreak, we set the values of *V*, $I_{HC}$, $I_{AC}$, and $D_{HC}$ to 0. For simulation purposes, the initial values for $I_H$ and $I_A$ are estimated as in Table 3. Furthermore, *S*(0) is obtained by subtracting these initial values from the total population. These values were used to perform the relevant simulations and obtain the values of the parameters in Fig. 4 that were fitted to the real data. The solution curve in comparison to COVID-19 data is shown in Fig. 4.

**Table 2:** Monthly cumulative COVID-19 cases in Ethiopia from March, 2020, to April 2023.

Months	Cases			
	2020	2021	2022	2023
January	–	137650	465158	499531
February	–	159072	468727	499981
March	25	206589	469758	500564
April	130	257442	470568	500834
May	950	271514	472743	–
June	5846	276174	488724	–
July	17530	280365	492237	–
August	52131	308134	493190	–
September	75368	345674	493579	–
October	96169	365167	493960	–
November	110074	371536	494578	–
December	124264	420342	498001	–

**Table 3:** Initial values for state variables.

Variables	S	V	$I_C$	R	$I_H$	$I_A$	$I_{HC}$	$I_{AC}$	$D_{HC}$
Values	119663000	0	25	2	413333	206666	0	0	0

**Table 4:** Values of parameters in Equation 1.

Parameters	Values	Source	Parameters	Values	Source
$\gamma _c$	1.93768/month	Fitted	$\eta _1$	0.02486/month	Fitted
$\gamma _h$	0.011/month	estimated	$\alpha$	0.1/month	Fitted
$\rho$	0.25	estimated	$\delta _h$	0.016/month	^45^
$\psi$	0.0048/month	Fitted	$\eta _2$	0.0274/month	Fitted
$\xi$	0.1935/month	estimated	$\omega$	0.07/month	^46^
$\sigma$	0.01	estimated	$\varphi$	0.0003/month	Fitted
$\theta$	0.0121/month	Fitted	$\kappa$	1.22	estimated
$\epsilon$	1.385	estimated	$\delta _{hc}$	0.03851/month	Fitted
$\delta _c$	0.01511/month	Fitted	$\phi$	0.00067/month	estimated

### Numerical simulation and results

Numerical simulations confirm the results of the qualitative analysis of the sub-models and the co-infection model. The impact of parameters on the expansion and control of COVID-19, HIV/AIDS and their co-infection were assessed. With some exceptions, initial conditions in Table 3 and values of parameters in Table 4 are employed to attain simulations with Python 3.9.

**Figure 5:**
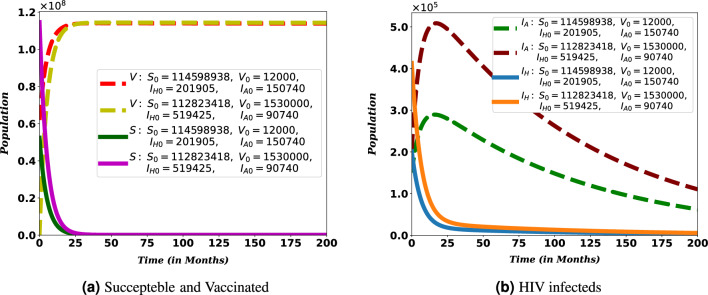
Global stability of HIV-free equilibrium for HIV sub-model.

**Figure 6:**
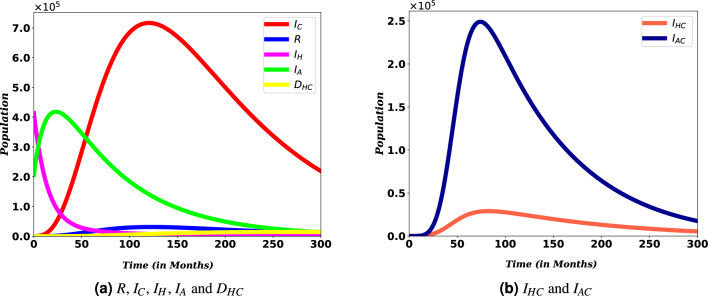
Local stability of disease-free equilibrium $E^0$.

The numerical simulation of the stability analysis of disease-free equilibria established in Theorem 4.5, and Theorem 4.7 are depicted in Figs. 5 and 6, respectively. These figures are used to show how the models, the HIV/AIDS sub-model and co-infection, behave about the disease-free equilibria. The parameter values $\gamma _h = 0.00502$ and $\omega = 0.15$, so that ${\mathscr {R}}_{0h} = 0.459473$, with different initial conditions, are used to show the global stability of HIV/AIDS-free equilibrium (see Fig. 5). On the other hand, $\gamma _c = 1.2$ and $\gamma _h = 0.008$, so ${\mathscr {R}}_0 = 0.791337$, are used to show the local stability of disease-free equilibrium of the full model.

#### Transmission rates versus co-infection cases

**Figure 7:**
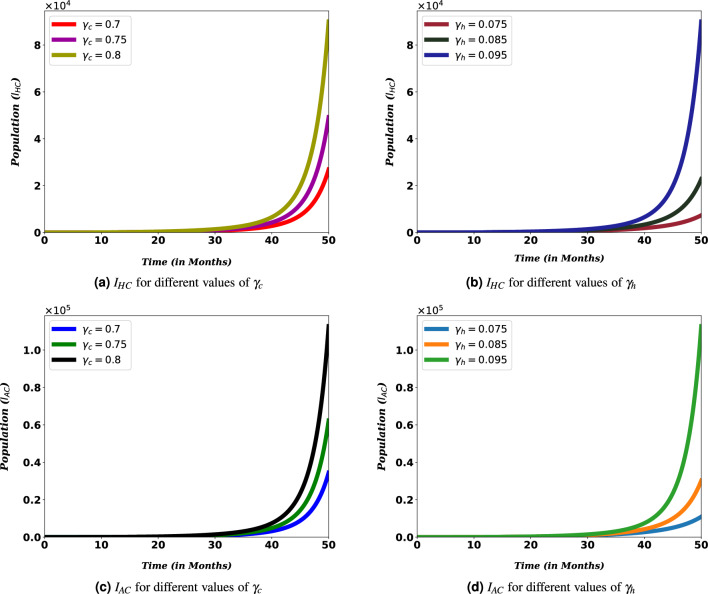
Simulation of $I_{HC}$ and $I_{AC}$ for different values of transmission rates $\gamma _c$ and $\gamma _h$.

In Fig. 7, various transmission rates are used to illustrate how they affect the number of co-infected populations. These sub-figures show that as transmission rates decrease, the value of the graphs decreases. An increase in $\gamma _c$ from 0.7 to 0.8 and $\gamma _h$ from 0.075 to 0.095, co-infection cases decrease faster than the higher value.

**Figure 8:**
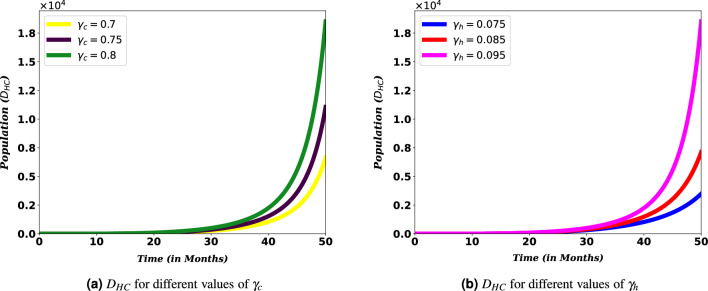
Simulation of $D_{HC}$ for different values of COVID-19 and HIV transmissions rates, $\gamma _c$ and $\gamma _h$.

Moreover, Fig. 8 show the contribution of HIV and COVID-19 to the population in $D_{HC}$. From Fig. 8a, we can see that it is essential to bring down COVID-19 transmission rates to reduce the potential for death from the burden of both diseases, HIV and COVID-19.

#### HIV transmissibility and immunosuppression

**Figure 9:**
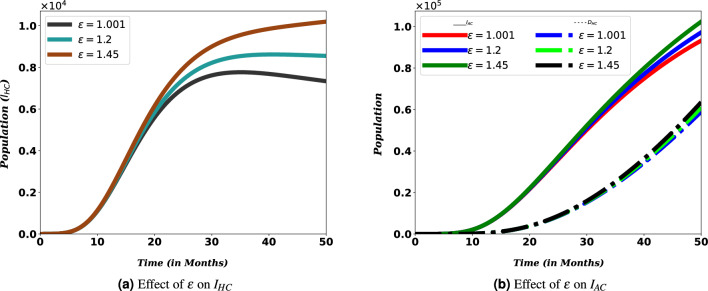
Effects of $\epsilon$ on the number co-infection.

**Figure 10:**
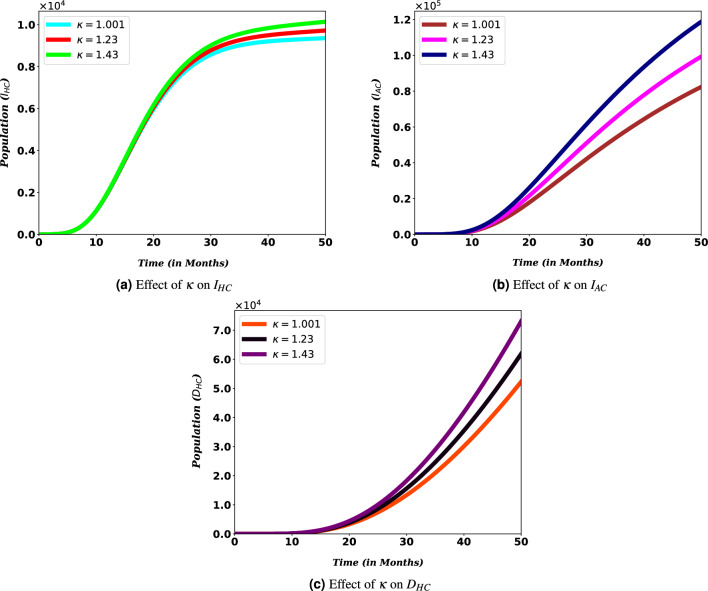
Effects of $\kappa$ on the number co-infection.

An increase in viral load in HIV/AIDS patients increases their ability to transmit the disease to uninfected people and makes them vulnerable to other diseases^6^. Increased HIV transmissibility and exposure to COVID-19 may also increase the number of co-infections. Figures 9 and 10 confirm this phenomenon. From these figures, it can be seen that COVID-19 and HIV/AIDS co-infection increase with increased HIV transmissibility.

Moreover, in Fig. 10a to c it is shown that HIV/AIDS immunosuppression increases the number of co-infection cases. That is, as the value of $\kappa$ increases, the population in $I_{HC}$, $I_{AC}$ and $D_{HC}$ also increases. One way or another, it can be understood that HIV/AIDS viral load is directly related to the likelihood of contracting COVID-19, which may contribute to the risk of co-infection-related deaths (see Fig. 10c). This implies that slowing the progression of the HIV/AIDS virus is important and critical to reducing the impact of COVID-19 and HIV/AIDS.

**Figure 11:**
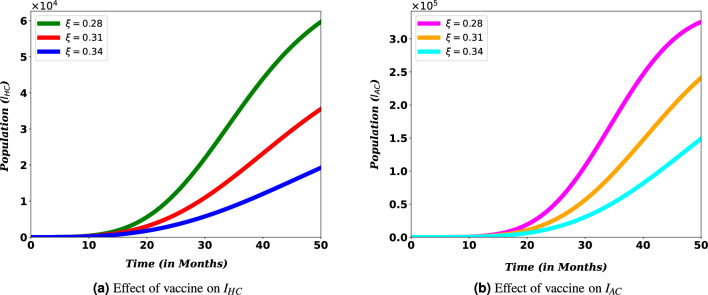
Effects of COVID-19 vaccination $I_{HC}$ and $I_{AC}$ population.

#### COVID-19 treatment and vaccine to minimize co-infection cases and risk of death among PLHIV

**Figure 12:**
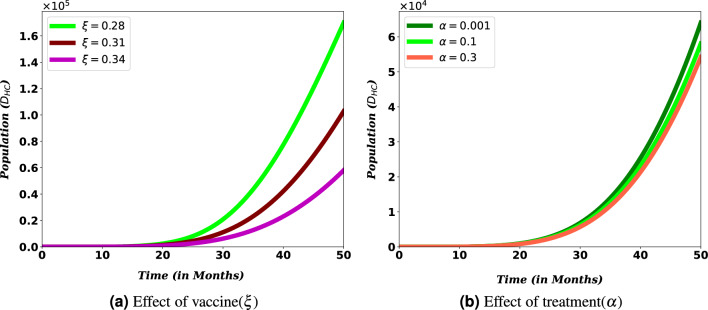
Effect of COVID-19 vaccination and treatment on $D_{HC}$ population.

The impact of COVID-19 vaccine on the number of co-infected populations is illustrated in Figs. 11 and 12. The sub-figures show that, as the value of the vaccination rate ($\xi$) of COVID-19 is increases, the number of co-infected populations decreases, which means the expansion of COVID-19 and HIV/AIDS co-infection will decrease.

**Figure 13:**
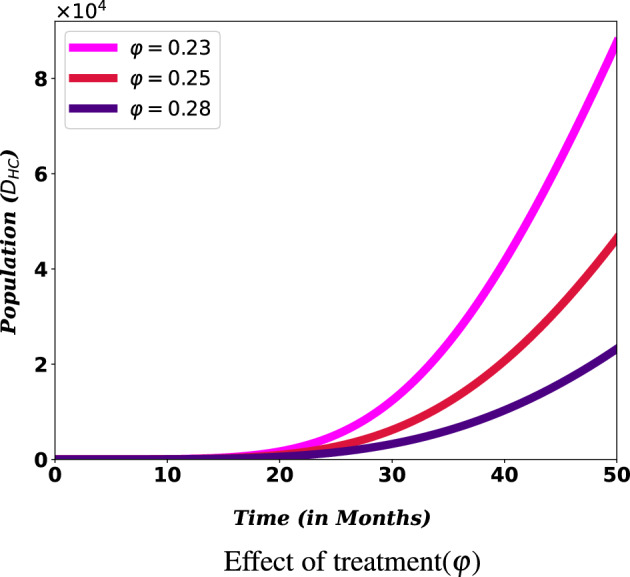
Effect of COVID-19 treatment, for individuals in $I_{AC}$ class, on $D_{HC}$ population.

For PLHIV, efforts such as vaccination minimize the chance of contracting COVID-19 disease, which might be one of the reasons for the relatively weak ability to produce antibodies and lower lymphocyte counts^47^. Treating co-infected individuals plays a vital role in reducing the number of deaths among HIV/AIDS patients. Simulations in Fig. 13 reveals that vaccinating susceptible and treating co-infected individuals have a reverse impact on increasing the death risk among PLHIV infected with COVID-19. This indicates COVID-19 diagnosis and treatment for HIV/AIDS infected people should be strengthened since it can lower fatalities from co-infection, especially when these people are vulnerable to the disease.

## Discussion

Although COVID-19 is under control with an incredible global public health effort, the public health impact of the disease in the community continues. People who have HIV/AIDS are among those who are affected by COVID-19. The mathematical models have been very beneficial in providing various reasons for the dynamics of disease and designing practical control strategies. In this study, we provide a co-infection model that governs the transmission dynamics of COVID-19 and HIV/AIDS in order to assess the impacts of COVID-19 among PLHIV and identify control strategies.

Transmission rates of the disease are the most important parameters that determine the dynamics of diseases, especially when these diseases claim the lives of many people. Considering the spread of COVID-19 and HIV/AIDS, they continue to pose a problem for the world. Figures 7 and 8 shows that the number of people coinfected with COVID-19 and HIV/AIDS cannot be reduced unless the rate of their transmission is made as low as possible. This demonstrates that the ongoing efforts to stop the two diseases must be stepped up. The same result has also been found in other similar study^34^. This observation is in direct agreement with mitigation approaches that aim at minimizing the transmission rate basically through self-protective measures (such as taking the vaccine and wearing masks for COVID-19 and abstinence, faithfulness, and protection for HIV).

HIV patients’ risk of transmitting the disease to another person or contracting another disease depends on their viral load. Following their compromised immunity, PLHIV may have an increased risk for severe disease from COVID-19 as well as hospitalizations^47,47^. This can be realized from Figs. 9 and 10. For people with HIV, when the ability to transmit the disease to another person decreases, the number of co-infected people also decreases, and when it increases, the number of co-infected people decreases. The same is true for the risk of exposure to other diseases. Along with this, if we look at Fig. 10, we can clearly understand that immunity has a significant contribution to the number of co-infected people. This is consistent with existing research and shows that more attention is needed for these people. In addition, it can be understood that the weakening of the immune system of HIV patients increases the death rate caused by COVID-19 and HIV/AIDS coinfection. These findings underscore the necessity to intensify efforts to provide HIV/AIDS patients with a treatment such as antiretroviral therapy, which serves two purposes for HIV/AIDS patients and their surrounding community: preventing HIV transmission as well as reducing HIV/AIDS patients’ vulnerability to other diseases.

The World Health Organization considers safe and effective vaccines to be a game-changing tool in controlling the COVID-19 pandemic^17^. This role of the vaccine can be indicated in Figs. 11 and 12. From the figure we can observe that covid-19 vaccination has a significant role on reducing the number of population. Especially it contributes a lot on reducing the risk of death due to the burden of these diseases. This result reinforces the WHO recommendation to prioritize HIV/AIDS patients during COVID-19 administration^18^. Therefore, there must be a solid effort to administer a vaccine to reduce the spread of co-infection and the death risk for HIV/AIDS-infected individuals. More over, Figs. 12 and 13 show that COVID-19 treatment has a vital role in reducing the risk of death due to the co-infection. More over, Figs. 12 and 13 show that COVID-19 treatment has a vital role in reducing the risk of death due to the co-infection.

## Conclusion

In this work, a deterministic model with nine compartments is used to describe the transmission dynamics of COVID-19 and HIV/AIDS co-infection. The basic properties of the model, positivity and boundedness of the solutions, are examined. That is, wellposedeness of the coinfection is established. To examine the models’ qualitative behavior, we first divided it into two sub-models, COVID-19and HIV/AIDS. COVID-19-free equilibrium is calculated and used to compute the controlled reproduction number (${\mathscr {R}}_{0c}$) which is given by: ${\mathscr {R}}_{0c}=\frac{ (1-\rho )\mu \gamma _c}{D_1 D_2} + \frac{\sigma (\rho \mu + \xi ) \gamma _c}{D_1 D_2}$. In the absence of vaccination, ${\mathscr {R}}_{0c}$ becomes ${\mathscr {R}}_{0c}^*=\frac{\gamma _c}{D_2}$. Since $\frac{ (1-\rho )\mu + \sigma (\rho \mu + \xi )}{D_1} <1$, We can see that vaccination reduces the average number of secondary infections produced by one COVID-19-infected individual during the course of the infection, which in turn reduces the number of coinfection cases.

Similarly, the basic reproduction number of HIV/AIDS-only sub-model is determined with the help of next generation matrix and is given by:${\mathscr {R}}_{0h}=\frac{\gamma _h }{D_4}\left( 1+\frac{\omega \epsilon }{D_5} \right)$. The first and second terms in ${\mathscr {R}}_{0}$ represent the number of new infections produced by one individual infected with HIV/AIDS during the time spent in $I_H$ and $I_A$ classes, respectively. The dominant reproduction number, from ${\mathscr {R}}_{0c}$ and ${\mathscr {R}}_{0h}$, is taken as the reproduction number of the coinfection model. The reproduction numbers serve as a threshold value for the dynamics of the system and the disease. Here are some of the main analytical findings of this study:

- The COVID-19-only sub-model has a locally stable disease-free equilibrium, whenever the associated reproduction numbers are less than unity. The biological implication of this finding is that a sufficiently small flow of infected people won’t cause an outbreak of the disease.
- The COVID-19-only model exhibits a backward bifurcation at ${\mathscr {R}}_{0c} = 1$. That is, in the neighborhood of 1, for${\mathscr {R}}_{0c} < 1$, a stable disease-free equilibrium coexists with two endemic equilibria: a smaller equilibrium (i.e., with a smaller number of infective individuals), which is unstable, and a larger one (i.e., with a larger number of infective individuals), which is stable. For ${\mathscr {R}}_{0c} > 1$, there are only two equilibria: the disease-free equilibrium, which is unstable and the larger endemic equilibrium, which is stable(see Fig. 2).
- The HIV/AIDS-only model has a locally and globally stable disease-free equilibrium whenever the associated reproduction number(${\mathscr {R}}_{0c}$) is less than unity. The biological implication of this result is that a sufficiently small flow of HIV/AIDS infected individuals will not generate an outbreak of the disease. It also has a unique endemic equilibrium whenever ${\mathscr {R}}_{0c} > 1$, i.e., the disease persists in the population whenever ${\mathscr {R}}_{0c} > 1$.
- The COVID-19 and HIV/AIDS co-infection model has a locally asymptotically stable disease-free equilibrium point whenever the dominant reproduction number is less than unity.
- From the analysis of the impact of parameters on reproduction numbers, it is realized that increase in COVID-19 treatment and vaccination rates has the adverse effect of causing more people to become co-infected. We also realize that the spread of the co-infection is accelerated by an increase in the two diseases’ transmission rates and the progression of the HIV infection.

The analytical findings are then verified by numerical simulations. The co-infection model was fitted to the real data of Ethiopia, and parametric values were estimated to further explain analytical results and determine the effects of parameters through numerical simulation. The following are some of the numerical results:

- The local and global stability of disease-free equilibria and backward bifurcation are illustrated.
- As COVID-19 and HIV transmission rates rise, so does COVID-19 and HIV/AIDS co-infection.
- Inhibiting HIV transmission and reducing HIV/AIDS patients’ vulnerability to other diseases are crucial for lowering co-infection cases and risk of death among PLHIV infected with SARS-CoV-2.
- Intensifying vaccination and treatment efforts reduces co-infection cases as well as the risk of death due to the burden of the co-infection.

From all these results, it’s understandable that vaccine administration and treatment efforts for COVID-19 must be strengthened to reduce the loss of lives caused by co-infection cases. Having such results will guide policy makers as to which specific protective measures should be implemented and focused on to reduce the loss of lives among people with HIV/AIDS.

There are several ways this study can be extended. The model does not consider COVID-19 asymptotic individuals who transmit the disease while contacting susceptible populations, so including their impact makes the model more representative. Self-protective measures are crucial in preventing the spread of viruses like HIV/AIDS as they cannot be cured. However, the specific contribution of self-protective measures is not taken into account in this study. Therefore, models that incorporate specific self-protective measures may be considered for advancing this research. This study can also be improved by incorporating optimal control strategies with different preventive and therapeutic strategies. Furthermore, the inflow of infected people due to the vertical transmission of HIV/AIDS and age structures can be considered in the areas of research for the future.

To summarize, our coinfection model have the potential to aid policy makers in devising more effective strategies for managing these diseases. It helps to figure out what factors affect the transmission of COVID-19 and HIV/AIDS diseases and what precautions need to be taken to prevent deaths due to the burden of these diseases. The fact that these diseases still have a detrimental effect on human health calls for further efforts to reduce or eliminate their effects on society.

## Data Availability

All data generated or analyzed during this study are included in this article.
